# Bilateral ampiginous choroiditis after COVID-19: a report of two cases

**DOI:** 10.22336/rjo.2025.67

**Published:** 2025

**Authors:** Doğukan Cömerter, Feyza Rümeysa Öz, Eyüp Düzgün

**Affiliations:** Department of Ophthalmology, Sultan Abdülhamid Han Training and Research Hospital, University of Health Sciences, Istanbul, Turkey

**Keywords:** ampiginous choroiditis, COVID-19, retinal inflammation, SARS-CoV-2, white-dot syndromes, AC = ampiginous choroiditis, APMPPE = acute posterior multifocal placoid pigment epitheliopathy, URTI = upper respiratory tract infection, COVID-19 = coronavirus disease 19, BCVA = best corrected visual acuity, OCT = Optical coherence tomography, FAF = Fundus autofluorescence, FA = fluorescein angiography, ANA = Anti-nuclear antibody, RF = rheumatoid factor, ACE = angiotensin-converting enzyme, MRI = magnetic resonance imaging, LP = lumbar puncture, CSF = cerebrospinal fluid, aPTT = activated partial thromboplastin time

## Abstract

Ampiginous choroiditis is a disease that includes features of both acute posterior multifocal placoid pigment epitheliopathy and serpiginous choroiditis. This article presents two patients who were diagnosed with ampiginous choroiditis in our clinic. A 28-year-old female patient presented to our clinic with blurred vision that had persisted for 2 weeks. Her best corrected visual acuity (BCVA) was 20/20. Her medical history included a COVID-19 infection about 1 month before. Fundoscopic examination revealed multiple grayish-yellow lesions with irregular borders and pseudopodial extensions, involving the posterior poles of both eyes diffusely. Fundus findings and multimodal imaging, including optical coherence tomography, fundus autofluorescence imaging, and fundus fluorescein angiography, indicated ampiginous choroiditis. The patient was treated with oral steroids and azathioprine. The second patient was a 34-year-old male under follow-up in the neurology clinic, with a diagnosis of transverse myelitis, who was referred to us for an ophthalmologic consultation. The patient had a COVID-19 infection a few weeks prior. The patient had no visual symptoms in our first examination, and his BCVA was 20/20. Fundus examination and multimodal imaging were associated with ampiginous choroiditis. The patient’s ongoing treatment was initiated by neurology, including oral steroids and azathioprine therapy. This treatment regimen was continued, and follow-up was conducted. In conclusion, these two cases suggest that the SARS-CoV-2 virus may serve as an immunogenic trigger for the development or reactivation of ocular inflammatory diseases in individuals predisposed to white dot syndromes.

## Introduction

Ampiginous choroiditis (AC) is a clinical entity that shares features of both acute posterior multifocal placoid pigment epitheliopathy (APMPPE) and serpiginous choroiditis (SC) [1]. Both APMPPE and SC can be observed in healthy individuals. It is assumed that both diseases occur due to the immune system attacking its biological structure because of the molecular and immunologic similarity between infectious and ocular antigens. Many etiologies have been suggested for SC, including autoimmunity, infection, vasculopathy, and degeneration. APMPPE is usually preceded by a flu-like illness such as an upper respiratory tract infection (URTI). It has also been associated with various infectious causes and vaccinations [2].

The new coronavirus, known as severe acute respiratory syndrome coronavirus 2 (SARS-CoV-2), is responsible for causing acute respiratory illness and is also associated with various systemic effects throughout the body. “Molecular mimicry” has been linked to the development of some systemic complications after coronavirus disease 19 (COVID-19), such as antiphospholipid antibodies, Kawasaki disease, and Guillain-Barré syndrome, similar to autoimmune choroidopathy. SARS-CoV-2 affects the microvascular system in many organs. The prevalence of ocular involvement is thought to be around 10% [3]. Casagrande et al. detected SARS-CoV-2 viral RNA in the retinas of three deceased patients [4]. In addition to retinal involvement, choroidal involvement has also been reported in individuals who had recovered from COVID-19. Kocamis et al. [5] claimed that COVID-19 caused disruptions in the vascular and stromal structures of the choroidal tissue, and another study observed thickening of the choroid, including the macular region [6]. Herein, we report two patients who were diagnosed with AC secondary to COVID-19.

## Case reports

### Case 1

**Fig. 1:**
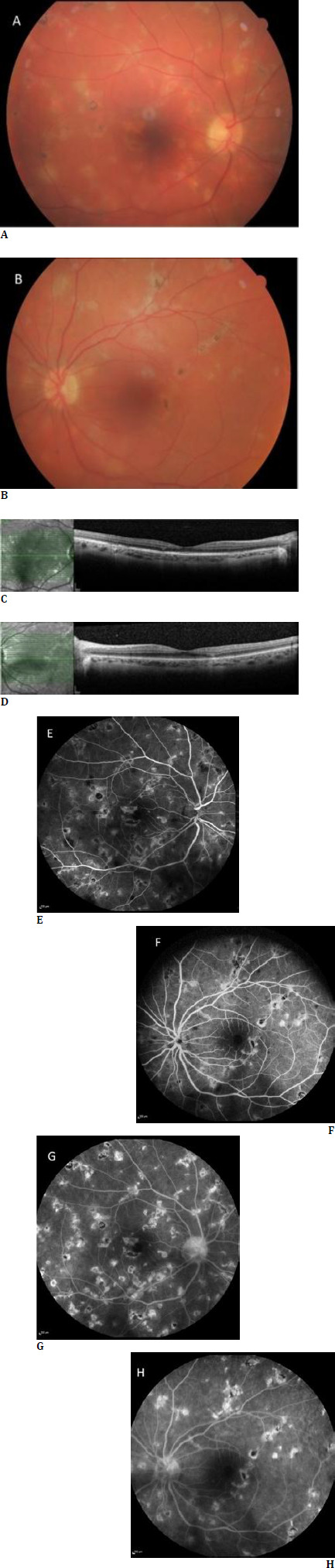
**A, B** shows Color Fundus Photography, multiple yellow lesions with irregular borders. The blurred images are associated with vitritis. OCT scans demonstrate defects in the outer retinal layers and choriocapillaris level in (**C, D**). Lesions appeared hypofluorescent in the early phase (**E, F**) and hyperfluorescent with irregular borders in the late phase (**G, H**) in Fundus Fluorescein Angiography

A healthy 28-year-old female presented with blurred vision that had persisted for 2 weeks. The patient mentioned that she had experienced fever-like symptoms (sore throat, runny nose, and headache), which started 1 month before the onset of visual symptoms. Still, she did not attend a medical center or receive any medical treatment at that time. She also stated that she had a history of exposure to people who were diagnosed as having COVID-19. The patient had no systemic diseases or any family history of these clinical symptoms. An ophthalmologic examination revealed a best-corrected visual acuity (BCVA) of 20/20 for both eyes. Intraocular pressure and eye movements were normal in both eyes. The anterior chamber was found to be clear, with no signs of cells or flare, but mild vitritis was detected in both eyes. A fundoscopic examination revealed multiple grayish-yellow lesions with irregular borders and pseudopodial extensions, diffusely involving the posterior poles in both eyes. Optical coherence tomography (OCT) scans demonstrated defects in the outer retinal layers and choriocapillaris levels. Fundus autofluorescence (FAF) imaging revealed that the lesions were predominantly hypo-autofluorescent, with a small number of lesions exhibiting hyper-autofluorescence. During fundus fluorescein angiography (FA), lesions appeared hypofluorescent in the early phase and hyperfluorescent with irregular borders in the late phase (**Fig. 1A-H**).

**Fig. 2:**
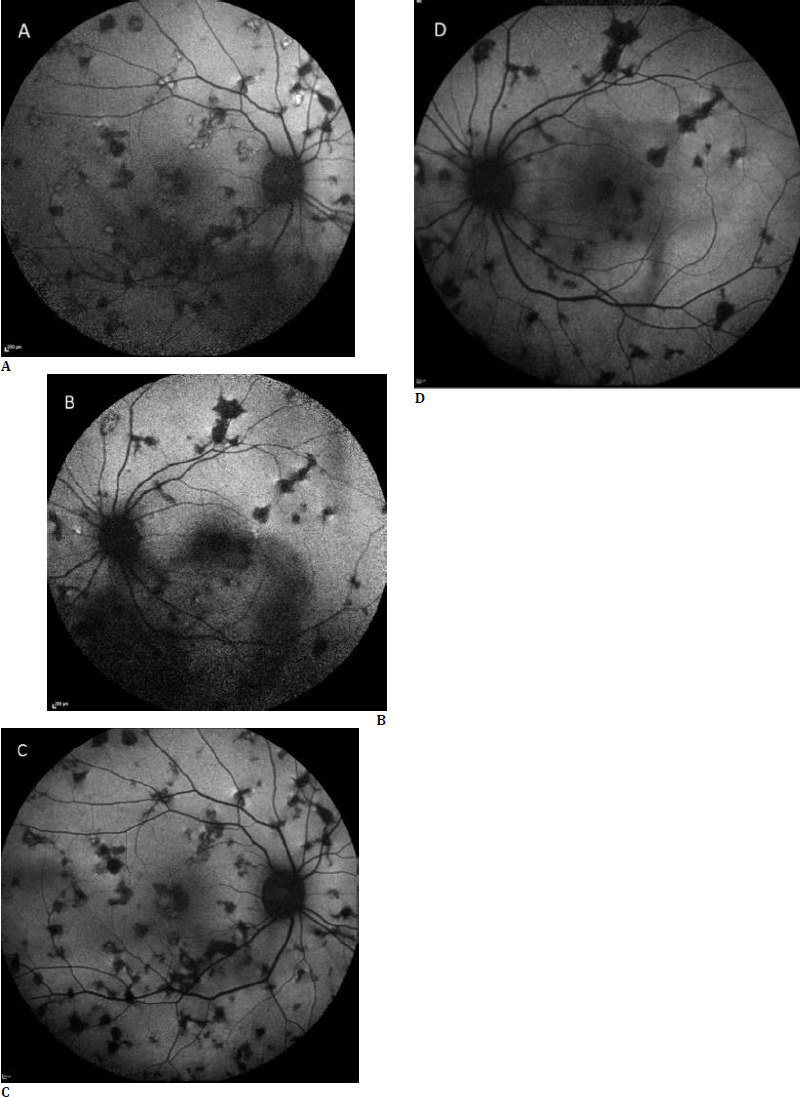
Fundus Autofluorescence. The lesions mainly appeared hypoautofluorescent, with a small number being hyperautofluorescent (**A, B**). After treatment, FAF imaging shows that hyperautofluorescent lesions have almost disappeared (**C, D**)

Laboratory tests showed normal levels of biochemical parameters. Serologic tests for cat-scratch disease, Lyme disease, and syphilis were negative. Anti-nuclear antibody (ANA), rheumatoid factor (RF), and angiotensin-converting enzyme (ACE) levels showed no significant elevations. The patient had no history of exposure to tuberculosis. In addition, the chest X-ray was normal. The QuantiFERON-TB Gold Plus test was negative. She had a negative tuberculin skin test with an induration of 3 mm. In the complete blood count, there was only mild elevation for C-reactive protein and erythrocyte sedimentation rate; all other parameters were at normal levels. The patient reported that she had not received a COVID-19 vaccination; therefore, COVID-19 antibody tests were performed, which revealed high levels of IgG antibodies for COVID-19 [Anti-SARS-CoV-2 S >2500 U/mL (negative <0.80 U/mL, positive >0.80 U/mL)]. This elevation is interpreted as suggestive of COVID-19-associated viral URTI. The multifocal pattern and angiographic features of the lesions supported the diagnosis of AC as a differential diagnosis. As for treatment, oral methylprednisolone (Prednol tablet, 64 mg) was started at 1mg/kg/day. At the first month follow-up, the patient reported an increase in blurred vision. In the biomicroscopic examination, vitritis persisted in both eyes. In response to this, azathioprine (Imuran tablets, 50 mg) was initiated at a dose of 1 mg/kg/day. The oral methylprednisolone was gradually tapered over a period of 3 months. Azathioprine was continued for 6 months and then tapered off. At the 24-week follow-up, both fundi showed no recurrences. When the patient’s 6-month FAF imaging was compared with the FAF images at the time of first admission, most of the lesions that initially showed hyper-autofluorescence had turned into hypo-autofluorescent lesions at 6 months, and no new hyper-autofluorescent lesions had developed. This indicated that the lesions had become inactive and there was no recurrence (**Fig. 2A-D**).

### Case 2

**Fig. 3:**
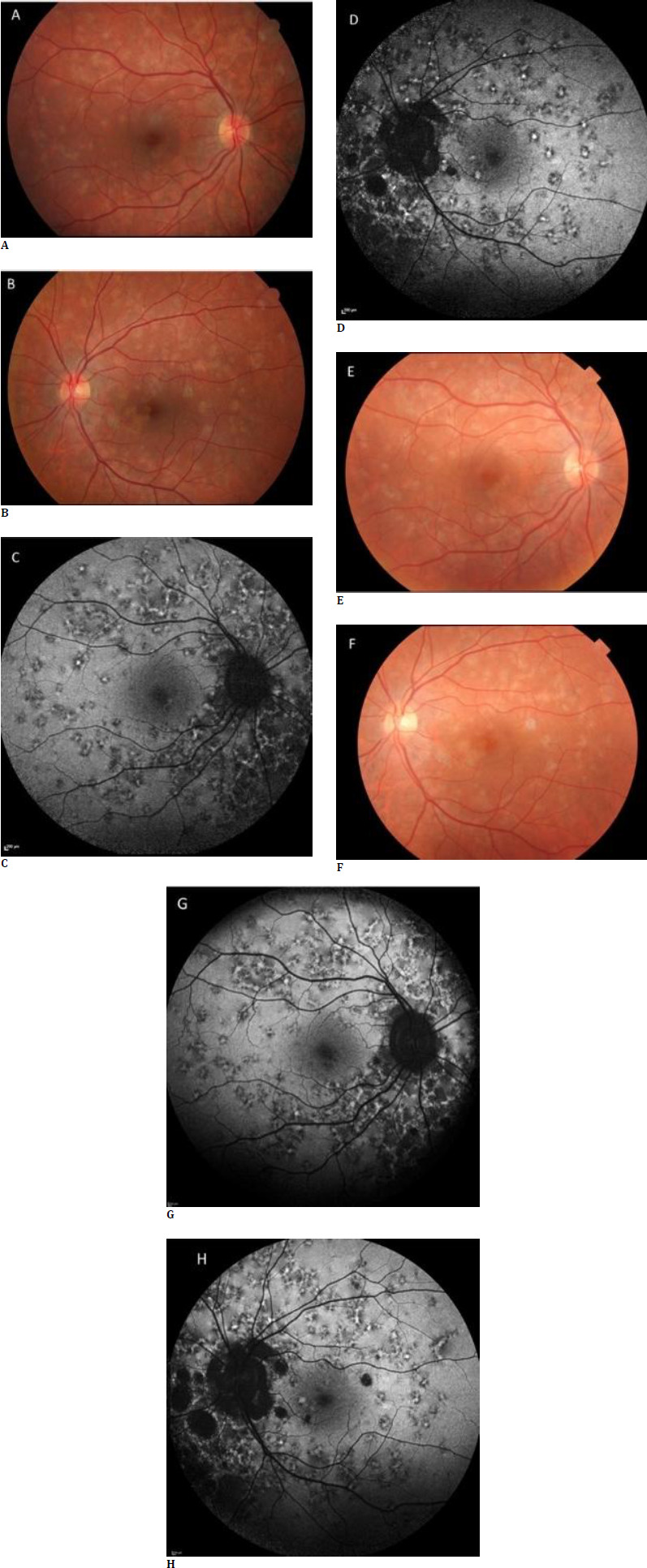
**A, B** Color Fundus Photography. Multiple diffuse yellow lesions at first presentation. **C** and **D** show the FAF images of the patient. **E** and **F** show Color fundus photography of the same patient at 12-month follow-up. **G** and **H** show that a few lesions became hypoautofluorescent, while the remaining ones were still active at the last visit

A healthy 34-year-old male presented to the neurology department with weakness and numbness in his lower extremities. During a detailed examination of his medical history, he mentioned that he had had a severe URTI 1 week before, and he did not seek any medical help. The neurology clinic ordered hematologic and biologic tests, which showed normal results. In addition, serology markers were negative. He had no history of systemic disease or tuberculosis, a normal chest X-ray, and a negative result on the QuantiFERON-TB Gold Plus test. Cranial and spinal magnetic resonance imaging (MRI) scans, as well as a lumbar puncture (LP), were performed by the neurology department. He had no ocular symptoms; it had been 6 weeks since his neurologic symptoms started before admittance to our clinic. An ophthalmologic examination revealed a BCVA of 20/20 for both eyes. Intraocular pressure and eye movements were normal. In biomicroscopic examination, the anterior chamber was normal with no signs of flare or cells. A fundoscopic examination revealed multiple grayish-yellow lesions with irregular borders and pseudopodial extensions, mainly involving the peripapillary region and posterior pole in both eyes. FAF imaging showed the lesions as mostly hyper-autofluorescent (**Fig. 3A-H**). During FA, lesions appeared hypofluorescent in the early phase and hyperfluorescent with irregular borders in the late phase.

**Fig. 4:**
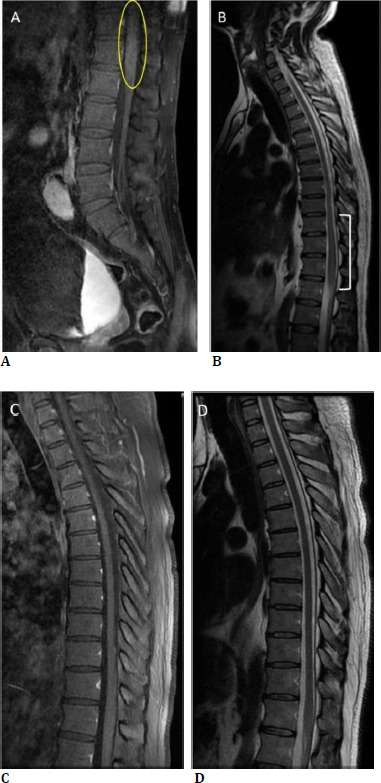
The patient’s first admission MRI images show enhancement (yellow circle) in the medulla spinalis on the sagittal contrast-enhanced T1-weighted image (**A**) and expansion (white line) in the medulla spinalis at the T8-12 levels on the T2-weighted image (**B**). After treatment, MRI images taken at 4 months appear normal on the sagittal contrast-enhanced T1-weighted image (**C**) and T2-weighted image (**D**)

In an LP evaluation, leukocytes were reported as 33/mm^3^ (lymphocyte 96%-neutrophil 3%). The LP opening pressure was 160 mm H2O. The cerebrospinal fluid (CSF) protein ratio was 0.67 mg/dL. There was no growth in the CSF culture, and HHV-7 DNA, HHV-8 DNA, and Anti-MOG IgG in CSF were also negative. COVID-19 antibodies were tested in both serum and CSF. Both tests were positive (Anti-SARS-CoV-2 S >2500 U/mL, Anti-SARS-CoV-2 S CSF: 44.88 U/mL, (negative <8.00 U/mL, positive >11.00 U/mL). Spinal MRI revealed an inflammatory appearance and involvement in T8-12 and conus medullaris, showing intense contrast (**Fig. 4A-D**).

Rheumatologic examinations were ordered to exclude the differential diagnosis of transverse myelitis. There was no elevation in ANA, RF, or ACE markers. Still, the lupus anticoagulant (LA) test was positive (44.80 sec), with the immunologic findings and progression pattern of these clinical symptoms indicating diagnoses of both transverse myelitis and chorioretinopathy due to COVID-19. The small and diffuse multifocal patterns of lesions observed in funduscopic examination, along with angiographic features, confirmed our diagnosis of AC. After evaluating the patient with the neurology department, the patient started oral Methylprednisolone (Prednol tablet 64 mg) at 1 mg/kg/day. Because the patient had spinal cord involvement, treatment with azathioprine (Imuran tablets 50 mg, 1 mg/kg/day was started, and acetylsalicylic acid (Coraspin 100 mg) was given for lupus anticoagulant positivity. Oral steroids were gradually tapered, and azathioprine was still being continued at the 12-month follow-up. At the patient’s most recent follow-up, most of the fundus lesions remained unchanged. However, some had transitioned to hypofluorescence on FAF imaging, indicating that they were still active (**Fig. 3A-H**). After treatment, the patient’s spinal MRI revealed that the contrast enhancement had resolved. The spinal cord had returned to its normal appearance (**Fig. 4A-D**).

## Discussion

Ampiginous choroiditis is an inflammatory chorioretinopathy with a high risk of visual impairment, sharing similar features with APMPPE and SC. Inflammation at the choriocapillaris layer causes ischemia, resulting in areas of non-perfusion or hypoperfusion in both the outer retina and choriocapillaris. Although the exact pathogenesis of the disease is unknown, similar processes have been observed after URTI or specific viral pathologies [1,2].

In this study, we present two cases of AC that developed after URTI due to COVID-19.

In the literature, we encountered several COVID-19-related chorioretinopathies (**Table 1**).

**Table 1:** Review of cases of COVID-19-related chorioretinopathy

YEAR	AUTHOR	CHORIORETINOPATHY	AGE	SEX	EYE	TREATMENT
**2021**	Tom, Elysse S et al.	Ampiginous choroiditis	25	Female	OU	Systemic steroids, Azathioprine
**2022**	Miyata, Manabu et al.	Punctate inner choroidopathy	25	Female	OD	Systemic steroids
**2022**	Providência, Joana et al.	Serpiginous choroiditis	41	Female	OS	Systemic steroids, Methotrexate
**2022**	Carvalho, Erika Moreira et al.	Ampiginous choroiditis	22	Male	OU	Systemic steroids
**2022**	Jain, Anupreeti et al.	Multiple evanescent white dot syndrome	17	Male	OU	Systemic steroids
**2022**	Nicolai, Michele et al.	Punctate inner choroidopathy	29	Female	OD	Systemic steroids
**2022**	Smeller, Lilla et al.	Multiple evanescent white dot syndrome	47	Female	OU	Topical steroids, topical cyclopentolate, periocular steroid injection
**2023**	Adzic Zecevic, Antoaneta et al.	Multiple evanescent white dot syndrome	40	Female	OS	Topical nepafenac and oral acetazolamide
**2024**	Ting, Michael et al.	Multiple evanescent white dot syndrome	69	Female	OS	Systemic steroids
**2024**	Seddigh, Sorayya et al.	Serpiginous choroiditis	28	Male	OS	Systemic steroids, Azathioprine
**2024**	Lund-Andersen, Casper et al.	Acute posterior multifocal placoid pigment epitheliopathy	17	Male	OU	Systemic steroids, Adalimumab

Carvalho et al. [7] reported that a patient who had COVID-19 confirmed through polymerase chain reaction (PCR) developed AC 7 days later. In another study, it was reported that a patient with similar fundus images had viral URTI findings 10 days before the onset of visual symptoms and that the COVID-19 antibodies turned positive within 5 weeks in the patient who did not undergo PCR testing because the acute period had passed [8]. Our first patient showed similarities to this case. The patient’s history of exposure to SARS-CoV-2-infected people, the patient’s lack of vaccination for SARS-CoV-2, and positive results of antibody titers, which were tested after 6 weeks, led us to a possible diagnosis.

It is known that after SARS-CoV-2 infection or COVID-19 vaccination, the virus can trigger autoimmune diseases due to its molecular similarities with the virus. Bowles et al. [9] reported that 56 patients with COVID-19 infections tested for antibodies, and 44.6% of them tested positive for LA. Furthermore, a relation between prolonged activated partial thromboplastin time (aPTT) values and COVID-19 infection was found. In coagulation tests, 91% of the patients with protracted aPTT tested positive for LA. In our second patient, we observed severe systemic complications associated with COVID-19. Besides the spinal cord involvement, LA positivity was also consistent with the literature. Although the patient’s aPTT result was close to the upper limit, it was evaluated as normal (aPTT = 38 sec).

It is crucial to distinguish between AC and APMPPE due to their differences in prognosis and treatment. APMPPE typically has a good prognosis with a self-limiting nature, whereas AC is characterized by recurrent attacks of inflammation that may lead to permanent vision loss. The presence of multiple, persistent lesions extending to the peripheral retina of both eyes, which was observed in both of our cases, supported our AC diagnosis. Besides, FAF imaging showed that lesions were still active in both eyes of the second case. Considering the non-healing nature of the lesions, a diagnosis of SC was added to our differential diagnosis; however, there were negative results for tuberculosis, and there was no history of exposure to tuberculosis, which ruled out this diagnosis. Based on these features, immunomodulatory treatment was administered in the first case due to persistent symptoms and findings. In the second case, considering the involvement of both the spinal cord and choroid by SARS-CoV-2, immunomodulatory treatment was already initiated. In addition, we observed punch-out-like peripheral retinal scars in our cases, which are observed in multifocal choroiditis. The overlap between multifocal choroiditis with panuveitis and other white dot syndromes has been reported in the literature [10].

## Conclusion

In conclusion, SARS-CoV-2 may act as an immunogenic trigger for the development or reactivation of ocular inflammatory disease in susceptible hosts, as seen in our cases, particularly those with white-dot syndromes.

## Data Availability

The data that support the findings of this study are available from the corresponding author upon reasonable request.
